# “It will sort of drive us to rethink our approach to high fat salt sugar products”- a qualitative analysis of businesses’ reactions to the landmark Food (Promotion and Placement) Regulations in England

**DOI:** 10.1186/s12916-025-04384-5

**Published:** 2025-10-21

**Authors:** Preeti Dhuria, Sarah Muir, Sarah Shaw, Wendy Lawrence, Emma Roe, Janis Baird, Christina Vogel

**Affiliations:** 1 Medical Research Council Lifecourse Epidemiology Centre, University of Southampton, Southampton General Hospital, Tremona Road, Southampton, SO16 6YD UK; 2 Primary Care, Population Science and Medical Education, Faculty of Medicine, University of Southampton, Highfield Campus, Southampton, SO17 1BJ UK; 3 School of Geography and Environmental Science, University of Southampton, Highfield Campus, Southampton, SO17 1BJ UK; 4 Southampton Biomedical Research Centre, National Institute for Health Research, University of Southampton, and University Hospital Southampton NHS Foundation Trust, Tremona Road, Southampton, SO16 6YD UK; 5 NIHR Applied Research Collaboration Wessex, Southampton Science Park, Innovation Centre, 2 Venture Road, Chilworth, Southampton, SO16 7NP UK; 6 Centre for Food Policy, City St George’s, University of London, Northampton Square, London, EC1V 0HB UK

**Keywords:** Food policy, Business perspectives, Qualitative research, Retail food environment, Less healthy foods and drinks, HFSS regulations

## Abstract

**Background:**

Retail food environments have largely become settings which promote less healthy foods to their customers. In an effort to prompt healthier choices, the UK Government introduced regulations in October 2022 restricting most retailers in England from promoting products high in fat, sugar, or salt (HFSS) at store entrances, aisle-ends, and checkouts, and their online equivalents. Evidence is needed on how businesses approach compliance and adapt to these regulations. This study used in-depth interviews to examine business responses and generate insights to support effective implementation.

**Methods:**

This cross-sectional qualitative study involved semi-structured interviews with 22 business representatives responsible for interpreting and implementing the regulations. The interviews were conducted via MS Teams/Zoom between August 2021–April 2022, prior to the implementation of regulations. Preparations to implement changes and predicted impact on businesses’ promotional practices were examined. Six researchers collected and analysed the data using an inductive thematic approach.

**Results:**

Participants’ reactions to regulatory compliance varied according to perceived commercial impact and resource availability. While some businesses explored opportunities for healthful promotions and invested in layout changes, a significant proportion planned to comply only to the letter of the law and were testing alternative strategies for unhealthy promotions. Trade bodies played a crucial role in preparations, supporting member businesses to interpret the regulations and fostering a unified approach to compliance. Anticipated barriers to compliance included challenges such as accurately assessing product scores, reformulating products to meet standards, and ensuring consistent store-level adherence within large businesses. To enhance the regulations’ impact, participants called for (i) smaller in-scope businesses to receive additional technical support from the government, (ii) manufacturers be required to share detailed nutrient information with retailers or a centralised product nutrient profile repository be established, and (iii) out-of-home businesses be required to comply.

**Conclusions:**

These mandated regulations hold potential to shift food retailers’ priorities from solely profit maximisation, to also supporting public health. However further government action is needed to ensure effective compliance for all business types and sizes. A consistent, long-term policy approach aligned with other food policies and informed by industry expertise to optimise implementation could better support obesity reduction.

**Supplementary Information:**

The online version contains supplementary material available at 10.1186/s12916-025-04384-5.

## Background

Obesity is a widespread societal issue and a risk factor for non-communicable diseases. Various social, economic, and food environment determinants [1] contribute to obesity prevalence and health disparities [2, 3]. Previous UK government food policies aimed at tackling obesity have primarily relied on voluntary shifts in business practices and initiatives promoting individual behaviour change. For example, voluntary reformulation schemes for packaged food manufacturers, such as the UK’s Public Health Responsibility Deal, encouraged companies to reduce salt, sugar, and fat content in their products but showed little progress in improving product profiles [4]. Previous public health campaigns, such as Change4Life, focused on raising consumer awareness of nutrition and encouraging healthier food choices but may have in fact increased dietary inequalities [2, 5]. Increasingly evidence suggests that voluntary measures and individually focused policies do not deliver widespread and sustained improvements in population health [6–8], reinforcing the need for stronger regulatory approaches to equitably address the causes of obesity and dietary inequalities [5, 9]. 

As part of the UK Government’s efforts to address the upstream determinants of childhood obesity, two mandatory food policies have been introduced: a levy on sugar-sweetened beverages in 2018 and restrictions on unhealthy marketing practices in retail outlets in 2022 [10]. Both policies aim to limit key drivers of purchasing by targeting elements of the 4Ps of marketing- product, price, place, and promotion [11]. The latter policy, the Food (Promotion and Placement) (England) Regulations 2021 (*hereafter the regulations*), restricts (i) multibuy promotions of HFSS foods and (ii) placement of HFSS foods at prominent locations including their online equivalents [12]. The restricted locations within retail settings, including checkouts, store entrances, and end-of-aisle displays, are clearly defined in the regulations [12]. The multibuy promotions ban was delayed until October 2025 because of government’s concerns about the cost-of-living crisis and the regulations’ impact on consumer affordability.[13, 14] The placement restrictions were implemented in October 2022 as they posed a less direct financial burden on consumers: Retailers that sell food (including supermarkets, franchise convenience stores, and non-food stores), with 50 or more employees and stores with sales area larger than 2000 square feet, must comply with these regulations. Speciality food stores, such as chocolatiers, confectioners, or cake stores, are exempt from location restrictions, but promotion rules will apply [15]. Specified products (i.e. those in scope of the regulations) include prepacked items that fall into one of thirteen in-scope categories (i.e. soft drinks, savoury snacks, breakfast cereals, confectionary, ice cream and lollies, cakes and cupcakes, sweet biscuits and bars, morning goods, desserts and puddings, sweetened yogurt, pizza, potato products, and prepared meals including products in sauce and breaded or battered foods) [16]. These products have been identified by policymakers as significant contributors to children’s excessive intake of calories and sugar, are heavily promoted, and are assessed using the 2004/2005 Nutrient Profile Model (NPM) [17]. Developed by the Food Standards Agency (FSA), this model assigns a nutritional score to prepacked foods to classify them as HFSS (i.e. less healthy products) or non-HFSS, thereby guiding regulations on food advertising on TV and retail promotions [17]. Points are assigned based on a product’s content of nutrients to limit (energy, sugars, saturated fat, and sodium) and nutrients or ingredients to encourage (fruits, vegetables, nuts, fibre, and protein). A final score is calculated by subtracting points for beneficial components from points for less healthy components. Foods scoring four or more points and drinks scoring one or more points are in scope of the regulations [12]. Food and drink items exempt from these regulations include unpacked foods within these 13 categories and those that form part of meal deals. Moreover, alcohol is not included, as the sale and promotion of alcoholic beverages are governed by “The Licensing Act 2003” and The Retail of Alcohol Standards Group in the UK [18, 19].

Removing less healthy foods from prominent locations in retail settings can reduce exposure to these options and has been shown to positively influence the healthfulness of consumer purchasing behaviours, thereby supporting public health goals [20–23]. Consumers from disadvantaged groups often face less healthful food environments, with lower availability, higher prices, and poorer placement and promotion of healthy foods, resulting in poor diets and diet-related health inequalities [24–26]. These consumers also show greater sensitivity to the effects of unhealthy food environments for a range of financial, social, and psychological reasons which makes it harder for them to access nutritious choices compared to consumers with greater personal resources associated with having higher educational attainment or income [2, 24, 27]. The regulations alter unhealthy retail food environments to facilitate healthier choices, while requiring less cognitive effort and preserving consumer freedom [28, 29]. Therefore, the regulations hold potential to support all consumers, regardless of socioeconomic status, by reducing the visibility of unhealthy options.

The primary policy objectives are to restrict the placement and promotion of HFSS foods to reduce impulse purchases and excessive consumption, both of which contribute to weight gain over time [30]. The regulations could lead to a decline in sales of HFSS products, which many businesses rely on for higher profits. As a result, companies may seek to reformulate products to avoid the regulations or reposition their HFSS promotions [31, 32]. Similar responses were observed when the Soft Drinks Industry Levy (SDIL) was introduced in the UK in April 2018. Many sugary drinks manufacturers reformulated their products or reduced portion sizes to lower the tax burden, which has contributed to a reduction in sugar consumption at the population level [33, 34]. These past industry reactions highlight the need to understand how businesses might respond to the Food (Promotion and Placement) regulations to help estimate their potential impact on the retail food system and public health outcomes.

Adopting a systems perspective, the perspectives of key stakeholders (consumers, local authority officers, businesses and public health experts) were examined rapidly in a related manuscript [35] to explore how the new regulations trigger multiple changes within food systems, particularly in the retail food environment. This rapid analysis enabled the identification of overarching themes and the development of key policy recommendations across all stakeholder groups to provide timely insights to policymakers [36]. The present paper offers a more detailed exploration of business stakeholders’ responses, examining the key factors and system interactions that are likely to influence business compliance with the new regulations. Business stakeholders affected by the regulations include retailers, manufacturers, and wholesalers [37]. Trade bodies also have an important role in food policy by representing the collective interests of their members and facilitate the dissemination of information between key stakeholders, such as businesses and governments [38]. Qualitative methods provide a methodologically advantageous approach by allowing in-depth assessment of stakeholders perceptions, preparations, and values.

This study addresses a critical gap in the literature and provides empirical evidence on how businesses adapt to, comply with, or may attempt to circumvent the new regulations. By exploring businesses’ approach to compliance, our findings offer valuable insights into implementation concerns and regulatory loopholes. Findings from this research can help to inform policymakers about how the regulations can be strengthened to maximise their intended public health benefits. The learnings from this research can be used to optimise implementation of the regulations in England and apply regulatory learnings across the devolved UK nations or other global jurisdictions. The specific research questions addressed in this study include the following: (1) How have businesses reacted to the introduction of the regulations? (2) What are the key recommendations from affected businesses for enhancing implementation and impact of the regulations?

## Methods

### Study design

Considering the complexity and interconnectedness of actors within the food system, this study utilised a systems approach and a cross-sectional qualitative design to obtain an in-depth understanding into businesses’ perspectives [39]. Ethical approval for this study was granted by the University of Southampton Faculty of Medicine ethics committee (Ethics ID-65419.A1). The study adhered to the Declaration of Helsinki, Research Governance Framework for Health and Social Care, Data Protection Act 2018 and the Consolidated Criteria for Qualitative Research (COREQ) recommendations (Additional file 1) [40].

### Study setting, population, and recruitment

Invitations to take part in the study were emailed to a diverse sample of *n* = 85 business stakeholders affected by the regulation in England, UK. A convenience sampling approach was undertaken to recruit business representatives who were willing to share their views. Efforts were made to obtain insights from people across the system who have knowledge or experiences of working within food retail or manufacturing businesses. Potential participants were identified from the following: (i) the research team’s existing professional networks, (ii) contact details publicly available from food businesses’ websites, (iii) and a list of business names who responded to Department of Health and Social Care (DHSC) implementation consultation (these data were obtained through a freedom of information request by the research team). Potential participants were also recruited through snowball sampling where participants introduced the research team to their colleagues. Following distribution of the initial email invitation (including participant information sheet detailing research and researcher background, and question guide), contact was made via email and/or phone (where available) to maximise participation. Potential participants who did not respond after four follow-up attempts over a month were recorded as non-responders (*n* = 63, 74%). Before the online interviews commenced, all participants agreed for their interviews to be recorded, completed a consent form and a short questionnaire (either via email or verbally) which provided information about their type of organisation, job title, expertise, and experience. Participants could withdraw from the study at any time.

### Data collection

Semi-structured interviews were used to collect data because they allow reciprocity in conversation and help the interviewer improvise questions based on novel information raised in interviewees’ responses [41]. Participants were made aware of researchers’ background and interest in improving public health outcomes. A conscious effort was made to balance participants’ perceptions and their construct of reality by embedding reflexivity within this experiential qualitative approach [42]. Researchers collecting data adopted a stance of refraining from exerting power as nutritionists and psychologists advocating for righteous food choices. Throughout interactions, researchers remained mindful of delays in the issuance of the government’s regulatory guidance, shifts in political priorities due to leadership changes, and the context in which the regulations were being implemented (post-COVID-19 pandemic, Brexit, and the early cost-of-living crisis). Participants appreciated the opportunity to openly discuss their experiences and provided detailed accounts of their preparations for the impending regulations. Interview data were collected over a 9-month period between August 2021 and April 2022. This period occurred before the release of detailed policy guidance (April 2022) and policy implementation (October 2022). Experienced qualitative researchers, PD (*MSc*), a registered public health nutritionist and SM *(PhD)*, a psychologist conducted and recorded the interviews using MS Teams or Zoom video conferencing software. Field notes were not made. Interviews were held individually or in pairs where two members of a business participated together. The interview guide (Additional file 2) was informed by a qualitative systematic review (*Dhuria, in preparation*) and discussions with the DHSC team responsible for the regulations. Questions asked about businesses’ preparations for compliance, changes to their promotional strategies, concerns related to implementing the regulations, support needs, and perceptions of potential unintended consequences. While 19 participants represented the views of their organisation, some participants (*n* = 3) preferred that their views were not linked to their organisation. Interviews lasted between 18 and 48 min. Interview recordings were transcribed verbatim and anonymised. All but three participants were unknown to the interviewers. Two of these three were collaborators in an intervention study [43] and one was identified through social connections of the researcher.

### Data analysis

Anonymised interview transcripts were uploaded into NVIVO software (version 14) for coding management [44]. Braun and Clarke’s six step process was adopted for the analysis [45]. PD led the analysis, initially familiarising herself with the data by reading the transcripts and making notes. Data were coded inductively identifying patterns of meaning against the research questions. The codes generated were clustered under four initial themes (i) businesses’ views of the regulations, (ii) businesses’ preparations to implement the regulations, (iii) impact on businesses’ profits and practices, and (iv) businesses’ role in supporting healthier purchases. An initial coding frame was developed to include research questions, themes, sub-themes, descriptions, and examples of data excerpts. WL, CV, and EM refined the analysis by reading two transcripts each, discussing the codes and reviewing the initial themes in the coding frame. SS double coded data from twelve participants to initial coding frame and shared her interpretations. PD refined the themes and discussed with the research team to develop richer interpretations of meanings. All researchers were women aged 30–63 years old with expertise in qualitative research, food policy, public health nutrition, psychology, and geography. The findings were discussed with a business representative (a retailer public contributor from a related study on the convenience store sector) to confirm the interpretation of the final themes. Based on their feedback, the sub-theme generated by the authors-'*Long-term coherent framework for food system transformation'-*was expanded to include greater consistency in government policy and action across all food environments, including the out-of-home sector and rapid delivery apps.

## Results

### Participant characteristics

A total of 20 interviews were conducted with 22 food business representatives across England. In two instances, two members of the same business took part together. Participant numbers against their organisation type are presented in Table 1.

**Table 1:** Participant organisations

Organisation details	Participants (*n*)	Job roles
Trade body (retail, wholesale, and manufacturing)	5	Director/Chief executive (4), Manager (1)
Supermarket	4	Senior manager (2), Manager (1), Nutritionist (1)
Convenience store	3	Owner (1), Manager (1), Coordinator (1)
Online retailer	2	Senior manager (2)
Non-food retailer^a^	1	Manager (1)
Manufacturer	6	Senior manager (3), Manager (3)
Wholesaler	1	Nutritionist (1)

^a^A non-food retailer refers to a business whose primary focus is not selling food

**Fig. 1:**
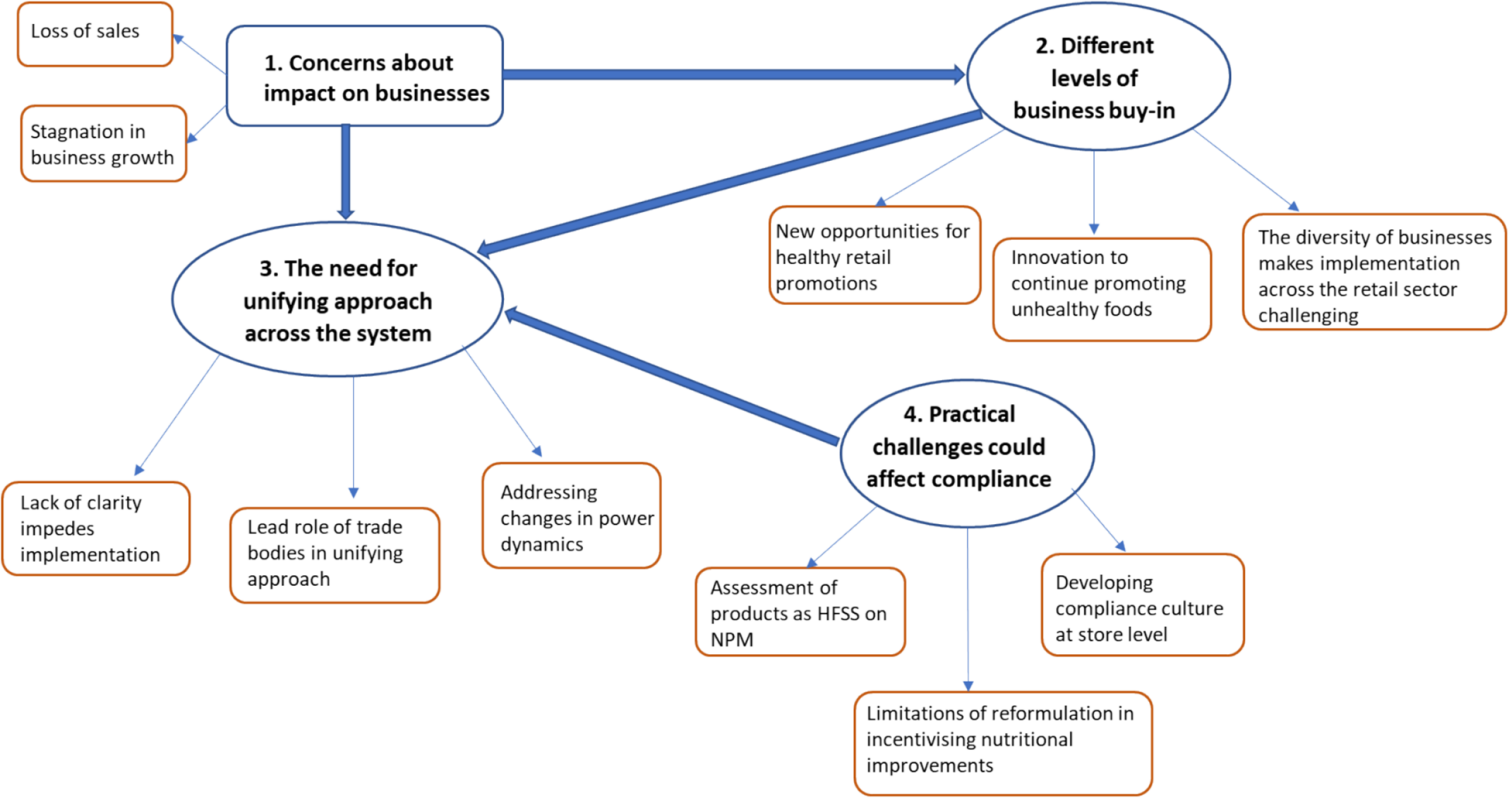
Businesses’ reaction to the regulations

Data analysis identified six main themes against the two research questions. Figure 1 illustrates the relationship between the first four themes that describe businesses’ reactions to the introduction of the regulations. Participants’ responses varied based on their concerns about how the regulations would impact them, leading to different levels of acceptance to these new rules. For many businesses, there was a progression from initial concerns and varying levels of acceptance to the recognition that a more consistent approach was needed towards compliance. Many participants pointed out practical challenges that need to be addressed to improve compliance, ensure fair competition, and unify the approach across the system. The final two themes, five and six, describe the need for proportional restrictions for different businesses and a pragmatic and consistent policy approach to improve population diets. Each theme is presented with participant quotes, using anonymised identification numbers and respective organisation to ensure confidentiality yet distinguish perspectives across business stakeholders.

### Research question 1: How have businesses reacted to the introduction of the regulations?

#### Theme 1: Concerns about the impact on businesses

##### Loss of sales

Participants noted that the regulations’ impact on businesses varied depending on their product mix and promotional strategies. They were worried that smaller in-scope businesses, which primarily sell HFSS products, would be disproportionately affected due to their limited range of food products, reducing their ability to adapt.

“So, it’s retailers who generally have a kind of a poorer customer demographic with less money available, [its] those who make a lot of their money through HFSS categories... they’ve not really wanted to engage with it. And I think it’s just because their entire business model is being affected by this.” - 11069 Manufacturer

Similarly, retailers with heavy reliance on generating profit-making sales on HFSS products through placement promotions at checkouts are likely to experience a notable decrease in sales due to the regulations. Consequently, they perceived the regulations as a substantial threat to one of their key revenue streams.

"So we as a business are very reliant on offering value through promotions and offering visibility through promotional space. Now obviously that goes. We also trade quite heavily from till point areas and that goes completely. So yeah, I do think from a financial perspective it will have a huge impact for us." - 11050 Non-food retailer

Participants felt that the regulations require them to work in ways that conflict with their profit-maximising marketing practices. The regulatory constraints reduce the freedom of their store staff to place products strategically to increase sales and achieve regional results.

"So, we’ve got an inherent problem here with getting to a good solution because they [companies] have to report to shareholders, shareholders want profits. So, we’ve got a mismatch between what we’re trying to do and what’s actually possible within these corporates." - 11088 Online retailer

##### Stagnation in business growth

Participants felt that the regulations disincentivise new product development. If manufacturers are not able to sell healthier versions of indulgent products that still fall within the regulation’s scope and get market share, they cannot invest in developing new products which will impact their business growth.

"I think for some manufacturers they just won’t be able to keep investing in product innovation." - 11039 Manufacturer

Participants also felt the regulations present obstacles for businesses planning to expand and may result in them limiting their number of employees to avoid falling within scope of the regulations.

"And it could be that actually some of those businesses don’t scale up, so they sort of stay stagnant and that could potentially prevent growth, to stay under the 50 [employees] thresholds." - 11088 Online retailer

Regulations may result in shrinking profits and inconvenience to customers as stores might need to close to carry out layout changes. There were concerns about the significant investment required for retail stores to comply with regulatory changes in terms of cost, time, and effort.

"I think between 25 to 30 [bays to] moves at a minimum. So that’s a lot of bays to move around to put say... three or four promotion ends and seasonal space into an aisle. So yeah, trying to work out the easiest solution and the quickest and less disruption to colleagues, customers. And then keep the cost down as well, it’s just not an easy thing." - 11056 Convenience store

Many retail businesses were already shifting their focus towards making layout changes in preparation for implementation of the regulations. As a result, these businesses were redirecting their financial resources towards refitting their stores to comply with the new regulations. This shift in focus had a negative impact on some manufacturers.

"[Retailer A] stopped everything with us last year and they’ve just re-informed us that their entire capital expenditure budget is going forward to pay for relaying [renovating] stores to meet the HFSS legislation, meaning they won’t be launching any new business with us for another year." - 11057 Manufacturer

#### Theme 2: Different levels of business buy-in

##### New opportunities for healthy retail promotions

Some participants viewed the regulations as an opportunity to reshape the retail environment to encourage healthier food choices. Their reactions indicated an understanding of broader objectives concerning obesity and public health. They saw the regulations as setting a new standard for product development and marketing, which prioritises healthier foods.

"It will sort of drive us to rethink our approach to high fat salt sugar products and undoubtedly, we will sell less [HFSS foods] and for us it’s about making sure that we do that in as sensitive a way as possible to both the business and the customers." - 11021 Supermarket

Some participants explained proactive initiatives were underway to reformulate products to ensure compliance with the regulations. They demonstrated a commitment to meeting regulatory requirements and a desire to compete commercially on healthier sales.

"So, what they [new product development team] are now thinking of is, if I develop this and it scores four or more, we’re not going to be able to put it on that permanent promotional offer. And that’s making them stop and think, can I develop something else?"-11011 Convenience store

Moreover, there was an acknowledgement of responsibility towards promoting healthier and more sustainable food options. Participants emphasised that these efforts should be integrated into corporate strategies and pursued collectively by various business stakeholders to create meaningful change throughout the food system.

"For us, one of the core sorts of aspects of our ESG [Environmental, Social and Governance] plan is to leverage the power of our brands to drive healthier, more sustainable choices. So, I think manufacturers and retailers must be responsible and do things the right way. They don’t operate in a silo." - 11039 Manufacturer

Participants felt that the regulations present an opportunity to make business sales data available for benchmarking and improving overall healthfulness of product scores. They advocated the government to encourage all businesses to set and achieve goals for enhancing the healthfulness of their product offerings and healthier sales.

"We are committed to annually report anyway as part of our commitment on how we do towards our own nutrition targets. So that’s something we report on annually and it’s audited…and I think anything that they [government] can do in terms of making data available." - 11091 Manufacturer

##### Innovation to continue promoting unhealthy foods

While some participants expressed intention to adhere both to the spirit and the specifics of the regulations, in many instances, commercial objectives were steering decisions solely towards compliance and finding innovative ways to continue promoting HFSS foods. Businesses were strategising ways to circumvent the regulations by promoting other high profit, often unhealthy, products from categories not covered by the regulations; many of which do not align with population health such as alcohol, vape products, fresh bakery products, and loose sweets.

"But we are looking at potentially doing stacks of bulk beer like the crates of beer or something like that. Instead of having them on the shop floor and just open the space up a bit…currently we have all our vaping behind but there will be like a display now on the shop floor..." - 11056 Convenience store

Businesses were also exploring various pricing strategies to bypass restrictions on multi-buy promotions, such as permanently reducing prices on unhealthy products. Many non-food businesses were considering using enhanced displays such as strategically placed shelves or stands to make HFSS products more noticeable and appealing to customers within aisles or other out-of-scope locations.

"We’ve also got some enhanced point of sale options to try and make the fixture stand out more because ultimately, we need it to become more eye catching, because there aren’t going to be the same number of disruption points around the store." - 11050 Non-food retailer

Manufacturers were also contemplating methods to enhance HFSS product packaging to ensure high sales volumes continued.

".... now it’s [HFSS product] going to be in an aisle, the packaging has to work so much harder because it has to stand out there against all the other unhealthy products in the aisle." - 11091 Manufacturer

One participant described developing communication packages to keep their customers informed about upcoming changes to guide them to the respective aisles where HFSS products would be located.

"We’re looking at building a sort of a communications package for how we communicate all of this to stores and to our customers and members. So, like making sure that they’re aware, why are we doing it, where we are doing it and then where we’ve moved [HFSS] stuff to. So, they can find what aisle to go to?" - 11056 Convenience store

##### The diversity of businesses makes implementation across the retail sector challenging

Participants shared that bigger businesses face a significant task of ensuring compliance across a large number of stores. However, they are better resourced on physical store space, financial capital and investments, and in-house teams to help them interpret and implement the regulations.

"I think that’s where we’ve seen in all our trials, in the bigger formats you can play around with your bargain bins, with your shelf space, your tills but in a small store that’s a lot more challenging to do." - 11001 Trade body

Participants also discussed difficulties in interpreting and implementing location restrictions both in-store and online. Each store and online equivalent required an individual assessment of its prominent locations and re-configurations that were complicated and open to interpretation.

"It’s not clear to us [how] to interpret the kind of physical, in aisle elements. So, I think some of the placement stuff for online businesses like us, where... we fall under some aspects of the definitions and not others and I think that’s where we’re struggling the most." - 11088 Online retailer

Participants discussed the additional challenges of making stores with dual entrances compliant, expressing concerns about the significant changes needed to the existing layouts across multiple stores before the regulations came into effect.

"So, we are going to have to do a lot of kind of re-layout within the actual main store. But it’s cutting across a lot of our main fixtures in a lot of our branches, which we’re realising is going to be quite a big job to redesign the store layout and implement that by next October." - 11007, Supermarket

"I think the definition of the main consumer route is fine for certain retailers, but for example Retailer 1, where you have a forced route through store, it’s led to so many questions." - 11069 Manufacturer

The inclusion of franchise businesses was deemed as unfair by some participants because many smaller stores are independent entrepreneurial businesses who do not receive significant resources or support from the Head Office.

"So essentially a business that on average employs eight people is now being treated like a business that employs more than fifty people in terms of compliance with the regulations. The Government have included them because they think that they get some support from Head Office to comply with the regulations, and they don’t." - 11037 Trade body

#### Theme 3: The need for a unifying approach across the system

##### Lack of clarity impedes implementation

Participants criticised the DHSC for delays in providing guidance on the regulations, which they felt weakened the policy’s impact from the outset. They expressed frustration over the lack of clarity, believing that the delayed guidance impeded the implementation process.

The difficulty is the way it has been managed and the fact that we’re what 6 to 8 months away from implementation and we still don’t know, we don’t have the guidance, there are literally hundreds of questions outstanding which DHSC haven’t answered." - 11011 Convenience store

Participants’ scepticism about the feasibility of enforcing the regulations effectively due to lack of clarity was evident across the interviews. They anticipated challenges in accurately assessing products as healthy/less healthy based on packaging information alone. Consequently, participants believed that enforcement efforts might be focused primarily on visible indicators, such as the presence of certain products like chocolates, rather than comprehensive product-based assessments. This perception influenced their attitude towards compliance, with some potentially not prioritising the regulations because enforcement was expected to be minimal.

"From everything I have heard from the enforcement community, they are having the same challenges we are having with interpreting the regulations. So, I think that it will be quite light touch. I’m not expecting to see a huge amount of improvement notices issued." - 11037 Trade body

On the other hand, some participants who were making changes in line with the regulations hoped for active enforcement to support a level-playing-field commercially. These participants, primarily from larger businesses that had invested resources to ensure compliance, expressed concerns that without rigorous monitoring and penalties, non-compliant competitors could gain an unfair advantage.

"It’s a shame because actually we need solid enforcement so that there is a level playing field again, right?" - 11012 Supermarket

##### Lead role of trade bodies in unifying approach

Proactive efforts were made by trade bodies to support their members in interpreting the draft guidance. They organised workshops, collaborated with local authorities, and engaged with DHSC, demonstrating a concerted effort to aid their members in comprehending and preparing to implement the new regulations. They developed guidance to identify in-scope and out-of-scope products and developed standardised approaches to define restricted locations. These resources made it easier for their members to understand the new regulations. However, businesses who were not members of these trade bodies missed out on this support.

"I’ve been doing one to one workshops with the majority of members to make them aware of what is coming their way. We’ve been having all of these conversations to be able to work out what the approach is likely to be. I’ve anonymously been able to share with the rest of the members some agreements that some of the bigger members have reached with the primary authorities [paid partnership with a single local authority to obtain regulatory advice]. For example, in terms of the scoring and obtaining information from suppliers and what due diligence looks like and how do we make sure that the classification is rightly done, etc." - 11004 Trade body

Participants described how they relied upon and valued the technical advice from their trade body on store layouts to ensure compliance with the regulations.

"Trade body B helped us determine a four-freezer configuration is fine, out of scope, it’s got no immediate end. Whereas if we had a six freezer or five freezers for example, in an island on its own, it’s got an identifiable aisle end. And is therefore classed as an aisle with an end." - 11057 Manufacturer

"There are still bits which I cannot fully understand. We have Trade body A, and we have the Trade body B, who are also helping us to translate that information. I think the way in which it’s been released as well has not been very well put together. Trade body A recently did a really good piece of work where they try to give an exhaustive list of inclusions and exclusions." - 11069 Manufacturer

##### Addressing changes in power dynamics

The regulations have the potential to shift power dynamics within the supply chain and affect the traditional roles and channels of influence held by each stakeholder. However, many participants also discussed the burden of communicating additional information between manufacturers, and retailers. Their concerns highlighted a need to address ongoing power dynamics to ensure the unified approach is feasible and sustainable.

"… attempting to start collecting the information from branded products, which is much more difficult, very slow going and a real challenge to the effective implementation of these guidelines, because there’s no absolute legal requirement on brands to supply the information." - 10021 Supermarket

Participants expressed concerns that potential reductions in volumes of HFSS products purchased would impact their ability to secure favourable deals with manufacturers. Additionally, participants highlighted that the regulations could affect supplier investments.

"Relationships with manufacturers are built over trading histories and volume. So, if our volume goes down that impacts the leverage that goes with that supplier. We also lean on suppliers for investments and if our turnover with them decreases, it will have a correlating factor with the investment that’s available from them." - 11050 Non-food retailer

#### Theme 4: Practical challenges could affect compliance

##### Assessment of products as HFSS on Nutrient Profiling Model

Participants were notably frustrated that businesses were required to score their full product portfolio in 13 categories against the NPM to ensure compliance. The lack of availability of a centralised NPM tool meant this assessment posed a challenge for many. Some businesses had developed in-house NPM calculators while others were outsourcing the product scoring process. The process of calculating product scores was particularly challenging for smaller businesses, who were less familiar with the NPM, struggled with incomplete nutritional data on product labels, or had limited resources to outsource the process.

"It’s [calculating scores] not straightforward even for companies that have in-house technical teams, it’s complicated and the current technical guidance has gaps. It requires information that’s not just on the label, like the amount of fruit and vegetable content, or the fibre content of a product that might not be on the label." - 11003 Manufacturer

Participants from business trade bodies described uncertainties about scoring a diverse range of foods where some products with unique ingredients or preparation methods may be classified as in-scope, while similar products may be exempt. For example, soaking raisins before incorporating them into a hot cross bun could potentially alter their nutrient profile as illustrated in the quote below.

"The calculation of fruit and veg is complex and depending on the type of product it is skewed one way or another. So, for example, if you leave the raisin soaking for longer, they might absorb more water before they go into a hot cross bun, so does it give you an advantage?"- 11004 Trade Body

##### Limitations of reformulation in incentivising nutritional improvements

Participants raised concerns that reformulating HFSS products may not improve their scores enough to fall out of scope, although small dietary improvements could have a positive impact at the population level.

"The shame of the nutrient profile model is it doesn’t encourage that [healthier versions] because I could convince my company to do a 30/40% sugar reduction or sodium reduction. Well, saturated fat reduction or an improvement in protein or whole grain fruits and vegetables, but the shifts required to unlock the nutrient profile model are almost untenable for most of our categories." - 11072 Manufacturer

Additionally, there was some apprehension that manufacturers might reformulate to produce exempt versions of ready meals that are ultra-processed and offer minimal health benefits.

"I would argue it’s [reformulations] likely to make food more processed and you could say that from a health and nutrition perspective, ultra-processed food is less healthy." - 11021 Supermarket

##### Developing compliance culture at store level

Participants expressed concerns about the challenges of training store staff to implement the regulatory changes, particularly given high staff turnover and limited nutrition knowledge among employees. They noted that compliance could not be managed solely by the Head Office and required ongoing staff training and monitoring.

"The people in the branches need to understand that if they’ve got gaps on their shelves, they can’t just fill it with anything anymore. All the right people are involved, and we are all having the right discussions but it’s how we implement those changes, at a time when, there’s a lot of change going on." - 11007 Supermarket

Businesses were conducting training sessions for their staff managers to build confidence and competence in implementing the required changes at the store level. However, there was heightened risk of going back to usual promotional practices, particularly during the festive season.

"We’re building a training programme. It doesn’t mean it’s going to all sink in and having that sort of thing where someone thinks they’re doing a good thing, say like Christmas time, we’ve always put those tubs of sweets stacked high at the front of the store." - 11056 Convenience store

### Research question 2: What are the key recommendations from affected businesses for enhancing implementation and impact of the regulations?

#### Theme 5: Proportional restrictions for different businesses

##### Enhanced support for smaller businesses

The regulations land differently for different businesses therefore a one-size-fits-all approach was not considered practical. Many participants asked for the government to provide appropriate resources and support to help smaller businesses with the complexities of scoring products and implementing layout changes within stores.

"I think there needs to be more support to help companies to do this, and particularly for smaller businesses who don’t have their own in-house technical teams." - 11003, Trade body

Additionally, they advocated for incentives that would encourage businesses to reformulate and innovate, thereby expanding their range of compliant products.

"The biggest thing that the government could do now is really support food manufacturing, and particularly small food manufacturers who don’t have the technical resources. They should be supported to both understand and implement the regulations." - 11021 Supermarket

Participants also emphasised the need for ongoing support to reduce confusion and address practical issues for retailers, manufacturers, and enforcement officers.

"Even though they publish a set of guidance, it doesn’t stop there. There’s going to have to be continuous available support on their side, and you know, for this piece of legislation. It’s a big one ... we need a dynamic process through which we get answers to these questions … we need some commitment." - 11004 Trade body

##### Collaborative approach to nutritional information

Participants highlighted retailers’ dependence on manufacturers to supply accurate nutrient information for scoring products effectively. They recommended that during the policy refinement process, the updated guidance should explicitly define the responsibility of brand owners and manufacturers to share correct and comprehensive nutritional and ingredient information and NPM scores.

"And if there was an onus on branded suppliers to be liable for supplying the correct information for their products as opposed to making the liability solely on the shoulders of retailers. It’s not just about the fact you sell them in your shop. The people who are responsible for the product need to take responsibility for their product." - 11021 Supermarket

Participants also proposed that the government could significantly enhance nutritional data management by creating a central repository of NPM scores for products. They recommended that the policy refinement process considers establishing a repository that could promote transparency and enable consistent scoring.

"Well, I think we are all calling out for a sort of, the government needs to kind of find a way of maybe centrally holding information about nutrient profiles for products. You know, like there’s the kind of composition of foods they had set that is held." - 11007 Supermarket

Moreover, having access to a comprehensive, centralised data repository can significantly impact business decisions and competitive dynamics. It could enable more informed business decisions regarding product assortment and promotions, ultimately enhancing the ability to impact overall product NPM scores and category management strategies.

"If these data were readily available across all products, if we had a view on the category scores, there’s so much more we can do to influence their [retailers] overall health scores. Get them [retailers] to understand their promotional decisions. Every aspect of category management could be influenced if we had better data." - 11091 Manufacturer

#### Theme 6: A pragmatic and consistent policy approach to reduce obesity

##### Extend restrictions to additional settings

Participants felt that implementing restrictions just in the retail sector would not be sufficient to improve population food choices and health. They emphasised the importance of addressing high calorie out-of-home products and extending the regulations to the out-of-home settings such as restaurants, cafes, and takeaways and grocery delivery apps to maximise impact on population diet and obesity levels.

"So obviously they [out-of-home sector] do have the free drink refill restrictions, but that’s obviously quite a marginal restriction compared to the full array of promotional restrictions for the retail sector, especially as we know that calorie intakes are much higher in the out-of-home sector." - 11003 Trade body

"You’ll have like rapid delivery apps, third party apps, where does the liability sit with that? I think it’s the retailer, but some of them there won’t be a retailer, this is like a dark hub. You know, there is no shop." - 11043 Trade body

##### Long-term food policy framework for food system transformation

Participants discussed the challenges businesses face in navigating various nutritional regulations and managing different criteria. They asked for the creation of a harmonised system where retail policies are joined up and inform businesses’ long term investment plans. For example, where improvements are made to a product’s nutritional value through ingredient reformulation, this could be directly linked to benefits to in-store product placement, and compliance with other policies such as nutrient packaging claims, and reduced taxation.

"I think what we would really love to see is just better consistency across policies…. for business it’s actually really a minefield to manage, you know, here’s the HFSS score now we’ve got everyone quite familiar with that, oh, but there are these reformulation targets, they don’t really relate, but OK, you still need to do that. Then if you want to make a claim on a product to be less sugar or higher in fibre, here’s a different set of criteria, it’s actually really complex. And it could be so much simpler to get to a much more meaningful outcome." - 11012 Supermarket

Participants recognised that the current traffic light front of pack labelling systems are not effectively aligned with the regulations. They were however optimistic that new regulations could improve food labelling in the UK, particularly in relation to the NPM model.

"My hope is that this legislation, if this is successful, it’ll help to drive the conversation around labelling so that we have labelling that’s in line with the HFSS algorithm. Because at the moment there’s also a disconnect between traffic lights and that. And I think it’s just, it’s too confusing for UK consumers to make a good choice at point of sale." - 11069 Manufacturer

Participants suggested that the government should use pricing strategies to make healthier options as competitively priced as less healthy foods, apply choice editing to reduce the variety of HFSS foods, and implement portion control mechanics such as dedicated campaigns for calorie-restricted treats to encourage mindful consumption. They also recommended fostering partnerships between manufacturers and retailers and collaborating with wholesalers to rethink business models by using the regulations as a lever to make healthier products more visible, accessible, and affordable.

"You know, you want both of them [healthy and less healthy foods] to be essentially at the same price and as attractive as possible to buy. And the problem is, as this sort of healthy calorie restricted ready meals are highly expensive." - 11067 Online retailer

"I think at Retailer 3, they’ve got this sort of history of taking off some of the choices. We only sell fair trade bananas now, so you don’t get a choice whether you buy non fair trade or fair trade. I think we could do more in health in that sort of choice editing." - 11011 Convenience store

##### Leverage businesses expertise to shift population food culture

Participants emphasised the need for a broader societal shift toward prioritising health by increasing consumer demand for healthier food and drink options alongside implementing the regulations. Increased consumer demand could potentially drive retail competition for healthier sales and significant changes in the food retail environment.

"It’s an entire kind of mindset shift that needs to happen. You know, government incentives around being healthy. Just there’s a whole kind of sociocultural thing that needs to change. Almost the same as what happened with like, stop smoking, how it’s just kind of not the norm anymore. You know, healthier options would just be desirable, would be kind of what people want." - 11001 Trade body

Many businesses see themselves as key stakeholders in reducing obesity and asked the government to engage with them early and more effectively in developing retail interventions. By leveraging businesses’ expertise in effective marketing strategies, successful product reformulations, and consumer engagement techniques, many participants suggested that the government can design more impactful and practical policies.

"I think the Government needs to almost listen and learn from some of those initiatives [that businesses utilise]. Understand what drives the most consumer value, what really is going to move the dial. Because we all have to learn how to design and execute them [the interventions] well." - 11072 Manufacturer

Participants expressed interest in understanding how the regulations influence consumer behaviour and recommended that government collaborates with businesses to evaluate their impact on consumers.

"I would hope that when they do the review, they do actually get some retailer partners and use some of the tools that the retailers have to be able to track what this [the regulations] is doing for consumers." - 11069 Manufacturer

## Discussion

### Principal findings

The participants’ reactions to the regulations were shaped by concerns about potential sales losses because they could no longer place high margin HFSS foods in prominent store locations. The regulations were perceived as potentially hindering business growth by limiting innovation in new product development, requiring significant investment in layout changes, and causing a knock-on effect on manufacturers in the supply chain. While some businesses identified opportunities to promote compliant and healthier product ranges, and invested in layout changes and reformulation efforts, others intended to comply only minimally, adhering strictly to the letter of the law. To maintain profits, many businesses were also exploring loopholes and developing creative strategies such as enhanced product packaging for HFSS products to capture shoppers’ attention and influence their purchasing decisions. The diversity within the retail sector, encompassing differences in business size, online presence, product types, and franchise models, further influenced the extent to which businesses engaged with the regulations. The delay in issuing of the government’s guidance posed significant challenges for businesses, complicating their interpretation of the regulations, and raising doubts about how well the rules would be enforced.

Business stakeholders, including trade bodies and different retailers, collaborated to develop a unified approach to NPM product scoring and layout adjustments, with the intent of adhering to the regulations. Participants emphasised the need for enhanced support, particularly for smaller enterprises who are not trade body members, to help them with store layout changes, product reformulation, and ongoing practical support to ease the challenges of implementation. They advocated for shared responsibility in product scoring, suggesting that brand owners and manufacturers should be involved, alongside the establishment of a centralised database detailing products’ NPM scores to guide business decisions and foster competition on product healthfulness. Additionally, businesses called for a more holistic policy approach from the government, proposing an extension of the regulations to out-of-home settings and rapid delivery apps, plus the creation of a long-term, coherent policy framework that aligns existing policies on labelling, NPM scoring and food retail marketing practices. The government could also encourage retailers to promote healthier products and limit less healthy options through strategic choice editing. Policies could cap the shelf space allocated to unhealthy items or set minimum stocking requirements for healthier options [46]. Financial incentives, such as tax breaks for retailers promoting nutritious foods and higher levies on unhealthy items, could further drive this shift [47]. While balancing public health with commercial viability is crucial, this approach supports broader efforts to create healthier food environments. Businesses positioned themselves as key stakeholders in addressing food-related public health challenges like obesity and urged the government to leverage their expertise in influencing consumer behaviour when developing future retail food polices.

### Comparison with previous literature

The regulations have forced a shift in food retail practices in the UK, with many retailers and manufacturers adapting to new regulations by reformulating products, redesigning store layouts, and adjusting marketing strategies to emphasise healthier options. A report from the Institute of Grocery Distribution confirms that numerous companies have expedited reformulation efforts to swiftly develop compliant products following the regulations’ announcement [48]. Within a year, of regulations being implemented, brands like Goodfella’s and Dr. Oetker launched non-HFSS pizzas, and Tyrrells, Kettle Chips, and Walkers introduced compliant crisps [49]. This shift mirrors the reformulation changes observed following the introduction of the SDIL in the UK, where manufacturers innovated due to taxation policy [33, 50]. Similar trends have been observed internationally, with sugar-sweetened beverage taxes in Mexico, Seattle, and Poland driving healthier product formulations [51–53] and influencing marketing strategies [54]. Findings from previous literature and our study strengthens the case for mandatory regulations to reshape industry behaviour because voluntary policies and commitments frequently result in minimal or selective improvements [4, 20]. Stronger joined-up regulatory approaches which are continuously refined in line with latest public health evidence are essential to driving meaningful improvements in retail food settings which truly benefit public health.

The previous sugar-sweetened beverage policies primarily targeted manufacturers, whereas the Food (Promotion and Placement) regulations aim to improve food marketing in retail settings, thereby targeting both retailers and manufacturers. Product reformulation is generally expected to support public health goals through improved product nutritional profiles. However, some participants expressed concerns about increased level of processing and while others pointed to limitations of reformulation in incentivising significant nutritional improvements. Public health researchers have raised similar concerns, recommending comprehensive government food policies to enhance the overall nutritional quality of foods across various categories, while ensuring that their implementation is closely monitored to confirm they deliver the intended population health benefits [55, 56].

While the UK SDIL applies to only 15% of soft drinks (those containing 5 g or more sugar per 100 ml) available to UK consumers [33], the regulations under review in this study cover a much broader range of products and brands. Consequently, marketing strategies in response to the regulations will likely vary across product categories [57]. While some product categories will be able to focus on reformulation and use of health claims, others are likely to pivot toward digital marketing, attractive packaging, or alternative promotional strategies to maintain business profits and consumer interest in HFSS products in the face of the regulations. The UK government has announced tighter regulations on the marketing of less healthy foods, in an effort to address multiple facets of food marketing [15, 58]. Restrictions on retail price promotions will come into effect in October 2025, while the ban on advertising HFSS products on television before 9 pm and in paid-for online media has been delayed until January 2026 [59, 60]. Notable regulatory gaps will still remain including permanent price reductions, loyalty pricing and brand advertising [61]. The selective scope of existing marketing regulations means that unpacked HFSS products and other products affecting health (alcohol and vapes) can continue to be heavily marketed. Moreover, our findings suggest that businesses are leveraging alternative marketing tactics (such as in-aisle promotions, enhanced packaging, and strategic displays) to sustain the promotion of less healthy foods, potentially undermining the regulations’ intended impact. Additionally, the rapid evolution of digital platforms and influencer marketing [62] means that many emerging marketing methods remain outside the regulatory scope. By addressing these loopholes, policymakers could strengthen the regulations to more effectively protect children from exposure to promotions that encourage unhealthy eating habits.

Furthermore, the diverse nature of food businesses affected by the regulations means their responses will differ based on factors such as the role of HFSS products in their revenue, the size of the business, and available support to make changes. Smaller and medium-sized businesses often struggle with compliance due to factors like limited knowledge and resources [63]. Some businesses may be reluctant to engage with the regulations if communications are limited, making them feel the regulations are being imposed on them as identified in previous UK research of a policy that aimed increase access to fresh fruits and vegetables in small retail outlets [64]. Smaller businesses often lack upfront investment and capacity to quickly redesign store layouts, potentially putting them at a competitive disadvantage. This situation could result in unintended consequences, such as increased non-compliance, a heavier burden on enforcement authorities, or market consolidation where only the most adaptable or well-resourced companies succeed [63, 65]. However, diffusion of innovation is also likely as larger retailers and manufacturers adapt their practices, smaller business may benefit through direct influence, increased supply of healthier product ranges in the market or the need to stay competitive [50]. The need to maintain and grow profits is a key factor that can undermine adherence to the spirit of the regulations or even compliance. Unhealthy foods often yield higher profit margins due to lower production and ingredient costs, extended shelf life, and greater affordability per calorie compared to healthier alternatives [66, 67]. Additionally, their high palatability, convenience, and satiating properties further reinforces their predominant promotion by the food industry [68, 69]. Ongoing monitoring and research investigating how various business types respond to the regulations is needed to address the economic incentives driving the production and marketing of less healthy foods.

Our findings indicate that businesses are encountering practical challenges in applying the NPM to a wide range of foods within the categories included in the regulations. In the UK, NPM has been used for television advertising restrictions since 2007 [17] and more recently by local authorities to restrict outdoor marketing practices, including the Transport for London HFSS advertising restriction policy [70]. While the Transport for London policy incorporates a degree of flexibility through its exceptions process, allowing for case-by-case evaluations of whether certain products can be advertised if they meet specific criteria [70], this approach is unworkable for the Food (Promotion and Placement) regulations. A case-by-case process could undermine public health messaging, further complicate compliance, and enforcement, and introduce inequities by favouring larger companies with legal teams to submit requests for exemptions. Uniform national application of the regulations, over most retail spaces, aims to simplify compliance and maximise public health impact. Although the absence of an exceptions process may limit opportunities for businesses to promote reformulated products that still fail to meet the NPM score; these reformulated HFSS products should not be promoted in the first place because they do not align with the overarching goal of the regulations which aims to reduce exposure to less healthy foods. Food companies are likely to prioritise reformulation when it aligns with their ability to market and sell products. A bigger shift in the mindset of businesses is required to focus development of healthy food products. Regulations and reformulation strategies are likely to yield the greatest public health benefits when they prioritise meaningful nutritional improvements rather than minor adjustments aimed solely at meeting marketing thresholds.

Our research suggests that new national food policies can encourage collaboration among retailers, and suppliers, fostering a more integrated approach to implementing the regulations. As was evident in our study, previous research has highlighted the crucial role played by trade bodies both in influencing government regulations and acting as informal regulators by establishing industry norms and practices [38]. Understanding the dynamics and impact of trade bodies is key to coordinating industry behaviour and influencing regulatory compliance to promote public health.

Some retailers expressed concern about potential reduced investments from manufacturers, who may no longer pay “shelf rental” fees to promote unhealthy foods in high-visibility areas. With the UK food retail sector dominated by a few major retailers that largely control which products are placed in prominent in-store locations [71], the regulations mandate businesses to modify product placement practices, creating a retail environment that prioritises healthier alternatives. This shift also requires manufacturers to invest in research and development to diversify their portfolios and create healthier products. However, prioritising healthier reformulations entails significant risks and upfront costs, which could disadvantage smaller manufacturers with limited resources [71]. To support this industry-wide shift, the government should consider offering innovation funds and incentives, such as tax benefits, to encourage new product development, reformulation efforts and ensure broader compliance [72].

### Policy and research implications

While the regulations are encouraging, they may only produce incremental changes in healthier food choices rather than the substantial shift needed to significantly improve population diet and reduce obesity and related health issues. To achieve better public health results, government messaging should refine the focus of the regulations from only restricting HFSS products to promoting healthy or minimally processed products, such as fresh fruits, vegetables, and whole foods [56]. Our study also highlights the need for greater coherence food policies targeting obesity. The NPM was originally designed to restrict television advertising of less healthy foods during children’s programs but has since been extended to broader marketing regulations [73]. When an algorithm is repurposed beyond its initial design, a transparent, evidence-based review is necessary to assess its effectiveness and refine its criteria if needed. Additionally, the selective application of the NPM to only 13 food categories within regulations deviates from its original intent, creating inconsistencies that require critical evaluation. To enhance policy coherence and maximise impact, better alignment between the NPM and front-of-pack nutrition labelling (FOPNL) regulations is needed. NPM apply a specific scoring formula balancing the content of positive (e.g. fruits, vegetables) and negative (e.g. calories, sugars) nutrients and scores per 100 g of product [12]. Inconsistently, labelling schemes focus on individual nutrient thresholds (fat, saturated fat, sugars, salt) and assign colours based on healthfulness thresholds and display values per 100 g or per serving [74]. While both tools are aimed at promoting healthier food choices to consumers, they can provide different messages about the same product. For example, a granola bar may score low on the NPM and therefore be considered a healthful product but may have high sugar content per serving and have a red traffic light for sugars [75]. These differing approaches may lead to inconsistencies in nutritional messaging for consumers which underscores the need for greater harmonisation. To enhance consistency in assessing and communicating product healthfulness, a policy review with the aim of aligning thresholds, as has been done in France, would be beneficial for British consumers [73, 76]. The UK Government conducted a public consultation in 2020 to refine its FOPNL, considering new international front-of-pack labelling systems like Nutri-Score and warning labels. The report from this consultation is still pending publication [77].

Further policy coherence could be achieved through mandatory reporting of the NPM scores of food and drink products. While some participants of this study described that their businesses have already committed to setting goals and reporting their sales of healthier product offerings, there is no public repository of product NPM score or sales figures against NPM scores. Many investors recognise the need for publicly available data to support companies in shifting their product portfolios toward healthier options [78]. The previous UK government backed away from mandating reporting on HFSS/non-HFSS product sales, opting instead for voluntary disclosures. However, health advocates emphasise that mandatory measures are needed to ensure consistent monitoring across all food businesses [79]. As part of its 10-Year Health Plan, the current UK government’s requirement for large food companies to report on the healthiness of their products, alongside future mandatory targets marks a welcome step forward [80]. However, its effectiveness will depend on several key factors, including the following: (i) the clarity and robustness of reporting metrics; (ii) the transparency of disclosures; (iii) the enforceability of health targets; and (iv) sufficient resources for effective oversight and enforcement.

Business participants in this study positioned themselves as key stakeholders in addressing childhood obesity and expressed a desire to engage with government on future retail food policies. Their operational insights, particularly in shaping consumer behaviour and implementing retail interventions, may offer practical value for policy implementation, provided strong safeguards and accountability structures are in place to protect public health interests [81]. Limiting or prohibiting industry involvement in the development of policy options and policy details related to population diet and obesity has been recommended, most recently in the UK House of Lords “Recipe for Health: a plan to fix our broken food system” report [82]. Monitoring industry activity is important and public sector resources could be increased to monitor business involvement in policy development [83] given their undue influence on population obesity levels through unhealthy marketing, controlling supply chains and powerful lobbying efforts [84]. Our findings indicate that many business arguments align closely with well-documented corporate political activity (CPA) tactics designed to resist public health regulations [85, 86]. Businesses emphasis on compliance costs reinforces the narrative that food policies are excessively burdensome and impractical. Another notable tactic involves diverting attention toward the out-of-home (OOH) food sector, despite evidence from the UK government’s *Family Food* module indicating that the majority of food purchases occur through retailers [87]. This narrative may be a strategy that actively serves to deflect retailers’ responsibility for the widespread availability of unhealthy food products. Additionally, claims regarding the challenges supermarkets face in obtaining nutritional information from branded suppliers represent another form of diversion. Given their dominant and unelected position within the food system, particularly in developed countries [88, 89], the claim of limited leverage may seem unconvincing. Supermarkets are known to impose strict private standards on suppliers such as mandatory compliance, detailed reporting and frequent audits, thereby limiting supplier autonomy and increasing costs, and reinforcing their substantial influence over the supply chain [90]. However, our findings also suggest that contracts may not be easily amended prior to policy implementation and that this level of power with manufacturers is not uniformly applicable across all business sizes. In our previous analysis of local authority officers’ perspectives of the regulations, large retailers were perceived to be well equipped with dedicated compliance teams, financial resources and primary authority agreements with local authorities. These conditions make them well-positioned to appropriately interpret regulatory guidance and implement necessary changes [91]. Business representatives in this study also highlighted that medium and small retailers face significant constraints, including limited space, lower financial capital and less personnel than large retailers which makes it more challenging for them to navigate the regulatory requirements effectively and efficiently. Our findings suggest that smaller and medium businesses require additional support, such as a mandate for manufacturers to provide clear and consistent NPM data or access to publicly available databases, NPM calculator, and guidance tools, to help them accurately assess product compliance and effectively navigate the regulations. Strengthening these resources could be even more impactful when combined with expanded public health nutrition capacity across the affected food system including local authorities, retailers, and manufacturers to further support compliance and maximise the effectiveness of the regulations [92, 93].

Businesses operate with economic goals, while regulations aim to achieve societal goals, such as improving public health. Addressing and/or managing these divergent interests is crucial and can be facilitated through establishing mechanisms early on and increasing transparency of engagement [94, 95]. Openly considering this dynamic interplay between economic interests and regulatory frameworks is necessary to inform policy refinement, address potential loopholes, and develop mechanisms that improve the effectiveness of regulatory measures and encourage responsible business practices. Future studies should use systems perspectives to evaluate the regulations, including monitoring changes in sales of HFSS products, both prepacked and non-prepacked, in retail and out-of-home settings, to assess regulations' effectiveness and inform necessary refinements.

### Strengths and limitations

A key strength of this study is engagement with a diverse range of businesses, including manufacturers, supermarkets, convenience stores, non-food and online retailers, and trade bodies. By exploring their views and preparations for implementing the regulations, the study provides a comprehensive understanding of how different stakeholders influence each other and have navigated the new regulations. We aimed to gather a broad range of perspectives rather than compare the views of specific business sub-groups. While there is potential for participants’ responses to be influenced by the interviewer’s identity as public health researchers, the study team were mindful of the broader contextual factors influencing compliance and aimed to maintain a neutral stance throughout the interviews. Participants exhibited a willingness to be open and candid, as evidenced by their discussion of the challenges posed by the regulations and their varying degrees of commitment to regulations’ implementation.

The findings of this study are not representative of all business perspectives. Many who declined to participate may have differing viewpoints. Additionally, the small number of participants within each business sub-type may not fully capture the diversity of viewpoints. Individual personnel within the participating businesses, for example nutritionists, may have more favourable views towards the regulations. This study was conducted following the announcement of the regulations but prior to the release of detailed government guidance which informs practical implementation. The subsequent issuance of this guidance may have clarified interpretational issues and influenced participants’ views and practices. Although evaluating participants’ perspectives post-implementation might have provided deeper insights into their adaptations, this was not feasible within the study’s available resources and timeframe. Further investigation post regulation implementation is merited.

### Conclusions

Our findings show that collaborative efforts and strategic adjustments occurred across food businesses as a result of the first regulations to limit unhealthy marketing practices in retail settings. While some proactive businesses took the lead in regulatory compliance to position themselves as leaders in healthier retail practices, many others invested resources into alternative strategies to continue marketing unhealthy foods, and some faced significant challenges with implementation. The mandated regulations have the potential to reshape competition in food retailing, encouraging a shift from just profit-driven approaches to one encompassing both public health and profitability. However, achieving this potential requires policy refinement and increased support for smaller businesses through tailored financial, technical, or logistical assistance to ensure equitable implementation. To maximise impact, the regulations should be extended to additional settings, such as the out-of-home sector, and harmonised with other retail food policies to foster healthier innovation. Leveraging business expertise to enhance the appeal of healthier options and operational knowledge in policy implementation could help to shift population food culture. However, governments must remain vigilant about conflicts of interest, ensuring that the development of future policies align with public health objectives and drive sustained, meaningful improvements in population diet.

## Supplementary Information

Additional file 1. COREQ Checklist Dhuria Business perspectives

Additional file 2. Dhuria business interview guide

## Data Availability

The data for this study were collected by the research team. Anonymised data can be made available upon reasonable request to the corresponding author pending approval.

## Abbreviations

UK: United Kingdom
HFSS: High fat, sugar, or salt
NPM: Nutrient Profiling Model
COREQ: Consolidated Criteria for Reporting Qualitative Studies
DHSC: Department of Health and Social Care
