# Supplementary material for: “It will sort of drive us to rethink our approach to high fat salt sugar products”- a qualitative analysis of businesses’ reactions to the landmark Food (Promotion and Placement) Regulations in England
Source: BMC Med. 2025 Oct 21;23:576. doi: 10.1186/s12916-025-04384-5 (PMC12538778; doi:10.1186/s12916-025-04384-5)
Supplement: Supplementary file 2 — Additional file 2. Dhuria business interview guide [file 12916_2025_4384_MOESM2_ESM.docx]

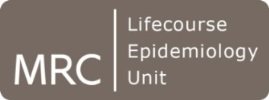

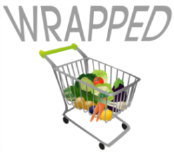


# MRC Lifecourse Epidemiology Unit

Southampton General Hospital

# Southampton S016 6YD

Telephone: 023 8120 4186


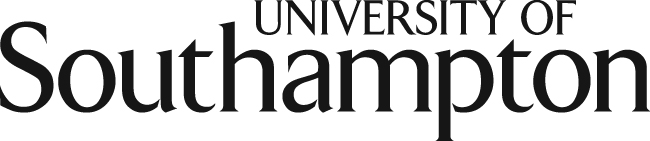


d d m m y y

**Store name:**  **Date**: T**ime:**

**Semi- structured interview guide - Businesses**

**Introduction**

Hello, I’m *[name]* from the University of Southampton. We are conducting research to understand your **views on the upcoming UK government legislation restricting the promotion and placement of unhealthy foods** in retail outlets.

For this research we will be asking some questions to understand your views as a business. It is likely to take approximately 30 minutes.

I would like to audio/video record the meeting with your permission in case I need to refer back to your responses later in the study. Only myself and the small team of researchers working on the study will have access to the audio/video file. Any information you give will stay confidential.

Taking part in this research is completely voluntary. You can choose not to answer any questions as we go along. You can stop taking part at any time, without giving a reason.

We really value your contribution. We plan to publish our anonymised findings and share these with policymakers to provide businesses’ perspective in terms of benefits and concerns related to this legislation.

Can I please complete the consent form with you? *[Complete consent form Appendix X]*

**Prompting questions**

**Views about upcoming legislation**

1. **What is your business’ current practice for promoting products in-store?**
   1. **How has it changed in recent years?**
2. **What is your opinion on the upcoming legislation?**

*Prompts*

- *How clear is the upcoming legislation?*

1. **How do you foresee the legislation being enforced?**
2. **In what ways has your business started preparing for the upcoming legislation?**

*Prompts*

- *Changing in-store layout/online layout*
- *Testing the impact legislation may have on your business*
- *Challenges in complying with the legislation*

1. **What changes will need to be made on your in-store/ online promotional strategies?**

*Prompts*

- *Changes in range of products on multibuy promotions*
- *Changes in the type of product being promoted in prominent places*
- *Altered promotion and placement of products deemed unhealthy by the legislation definitions*

1. **What preparations have you made with your suppliers/manufacturers?**

*Prompts*

- *Impact on relationships with manufacturers of high fat, sugar and salt products*

1. **What concerns do you have about the impact of the legislation on your business?**

*Prompts*

- *Impact on sales/ profits both online and in-store*
- *Impact on existing or new customers*

1. **Are there any areas where you feel your business could benefit from government support?**

*Prompts*

- *Support from local enforcement officers?*

1. **What unintended impacts do you foresee could result from this legislation?**
2. **What do you think the benefits of the legislation will be?**

*Prompts*

- *Possible benefits* *for customers, businesses, society, government*

**Perceptions about the role of businesses in supporting customers to buy more healthy products**

1. **More generally, what do you think is the role of businesses in supporting customers to buy healthier products?**

*Prompts*

- *Role of personal responsibility*

1. **What opportunities are there for your business to promote healthy eating more?**

*Prompts*

- *Increasing prominence of F&V, wholegrain products in stores/online*

**End with:**

- What else would you like to share, or ask me about?
- Thank you for your time.
